# Development of machine learning model for diagnostic disease prediction based on laboratory tests

**DOI:** 10.1038/s41598-021-87171-5

**Published:** 2021-04-07

**Authors:** Dong Jin Park, Min Woo Park, Homin Lee, Young-Jin Kim, Yeongsic Kim, Young Hoon Park

**Affiliations:** 1 Department of Laboratory Medicine, College of Medicine, Ewha Womans University of Korea, Seoul, South Korea; 2 Department of Laboratory Medicine, St. Vincent’s Hospital, The Catholic University of Korea, Seoul, South Korea; 3 Department of Research, Future Lab, Seoul, South Korea; 4 Finance, Fishery, Manufacture Industrial Mathematics Center on Big Data, Pusan National University, Pusan, South Korea; 5 Department of Laboratory Medicine, College of Medicine, The Catholic University of Korea, Seoul, South Korea; 6 Division of Hematology, Department of Internal Medicine, College of Medicine, The Catholic University of Korea, Seoul, South Korea

**Keywords:** Experimental models of disease, Information theory and computation, Machine learning

## Abstract

The use of deep learning and machine learning (ML) in medical science is increasing, particularly in the visual, audio, and language data fields. We aimed to build a new optimized ensemble model by blending a DNN (deep neural network) model with two ML models for disease prediction using laboratory test results. 86 attributes (laboratory tests) were selected from datasets based on value counts, clinical importance-related features, and missing values. We collected sample datasets on 5145 cases, including 326,686 laboratory test results. We investigated a total of 39 specific diseases based on the International Classification of Diseases, 10th revision (ICD-10) codes. These datasets were used to construct light gradient boosting machine (LightGBM) and extreme gradient boosting (XGBoost) ML models and a DNN model using TensorFlow. The optimized ensemble model achieved an F1-score of 81% and prediction accuracy of 92% for the five most common diseases. The deep learning and ML models showed differences in predictive power and disease classification patterns. We used a confusion matrix and analyzed feature importance using the SHAP value method. Our new ML model achieved high efficiency of disease prediction through classification of diseases. This study will be useful in the prediction and diagnosis of diseases.

## Introduction

Machine learning (ML) has had tremendous impacts on numerous areas of modern society. For example, it is used for filtering spam messages from text documents, such as e-mail, analyzing various images to distinguish differences, and extraction of important data from large datasets through data mining. ML makes it possible to uncover patterns, construct models, and make predictions by learning from training data^1,2^. ML algorithms are used in a broad range of domains, including biology and genomics^3,4^. Deep learning (DL) is a subset of ML that differs from other ML processes in many ways. Most ML models perform well due to their custom-designed representation and input features. Using the input data generated through that process, ML learns algorithms, optimizes the weights of each feature, and optimizes the final prediction. DL attempts to learn multiple levels of representation using a hierarchy of multiple layers^5^. In recent years, DL has overtaken ML in many areas, including speech, vision, and natural language processing^1,6^. DL and ML are also increasingly used in the medical field, mainly in the areas of ophthalmology and speech^3,7^. The deep neural network (DNN) is a type of DL that uses multiple hidden layers^8^ and is renowned for analysis of high-dimensional data. As bioinformatics data are usually high dimensional, DNN may be a suitable model for bioinformatics research^2,5,9^. In addition to image and text data from medical charts generated in hospitals, various types of laboratory data must be analyzed, which are mostly composed of numbers. However, very few studies have applied DNN to structured numerical medical data.

In practice, the symptoms described by patients, physical examinations performed by physicians, laboratory test results, and imaging studies such as X-ray and computed tomography (CT) are generally needed to evaluate a patient’s status and diagnose a specific disease. However, little research has been conducted into the predictive power and accuracy that can be achieved using laboratory data alone for the diagnosis of specific diseases. Therefore, the purpose of this study was to develop predictive models that can be used by physicians to make decisions in the hospital setting based on DL and ML using laboratory data alone, and then to validate our model through comparison of its predictions with the diagnoses of physicians. In addition, we generated an ensemble of DL and ML models to improve performance. The Shapley additive explanation (SHAP) method^10,11^, which was recently developed, was used to determine the features that are important to each disease and to identify predictive relationships between diseases and features. In this manner, it is possible to obtain utility from laboratory tests that would not be conclusive otherwise.

## Results

### Performance evaluation of neural network

We developed a new neural network (DL model) and trained it using 88 different parameters (86 laboratory test features, sex and age). We validated our DL algorithm using stratified five-fold cross-validation. We applied the TOP5 criterion (five most likely diseases) for evaluation of the model, as the purpose of our study was to create an artificial intelligence (AI) model to assist physicians with diagnosing diseases. In addition, we assessed the performance of each model using F1-scores, because there was an imbalance problem in the number of each 39 diseases. The F1-score and accuracy of our DL model were 80% and 91%, respectively. Precision and recall were 77% and 87%, respectively, for the five most common disease categories in Supplementary Table S2.

Interestingly, the DL model showed a different result in prediction of disease categories compared to the two tree-based boosting ML models (LightGBM, XGBoost). The top 10 diseases in terms of F1-score for the DL model were tuberculosis pleurisy, acute hepatitis B, malaria, acute lymphoblastic leukemia, acute leukemia of unspecified cell type, aplastic anemia, scrub typhus, acute myocardial infarction, acute hepatitis A, and acute pyelonephritis, in that order in Table 1. In addition, differences in performance were observed between the DNN and ML models for prediction of 38 different diseases. That is, DNN showed higher prediction performance for specific disease categories (sepsis, scrub typhus, acute hepatitis A, other specified acute viral hepatitis, acute respiratory distress syndrome, liver abscess, and urinary tract infection (UTI)).

**Table 1:** Neural network (DL) model TOP 10 result using accuracy performance measure.

ICD 10 CODE(M)	Disease classification	N of data	Precision	Recall	F1-score	Support
A1B	Tbc pleurisy	76	1	1	1	15
B1B	Acute hepatitis B	11	1	1	1	2
B5	Malaria	15	1	1	1	3
C9A	Acute lymphoblastic leukemia	6	1	1	1	1
C9C	Acute leukemia of unspecified cell type	7	1	1	1	1
D6	Aplastic anaemia, unspecified	11	1	1	1	2
A7	Scrub typhus	100	1	0.95	0.98	21
I2B	Acute myocardial infarction, unspecified	794	0.98	0.97	0.98	160
B1A	Acute hepatitis A	208	1	0.93	0.97	45
N1A	Acute pyelonephritis	578	0.97	0.91	0.94	124

*M* modified.

### ML results (XGBoost, LightGBM)

We developed two ML algorithms using LightGBM and XGBoost. These algorithms were trained using 88 different parameters in the same dataset used with the DL model. We proceeded with the learning process, leaving missing values in place due to the possibility of bias when missing values are replaced with other values (mean, median, etc.). We validated our two ML algorithms using stratified five-fold cross-validation. For the LightGBM prediction algorithm, the F1 score, accuracy, precision, and recall were 76%, 91%, 73%, and 85% for the five most likely diseases, respectively in Supplementary Table S3. For the XGBoost prediction model, the F1 score, accuracy, precision, and recall were 78%, 93%, 76%, and 86% for the five most likely diseases, respectively in Supplementary Table S4. Using the F1-score criterion, LightGBM sequentially showed malaria, toxic liver disease with hepatitis, acute myocardial infarction, unstable angina, acute pancreatitis, liver cirrhosis, acute hepatitis A, diabetic ketoacidosis, end-stage renal disease (ESRD), and tuberculosis as having the 10 highest F1-scores in Supplementary Table S5. The XGBoost model sequentially showed malaria, toxic liver disease with hepatitis, acute myocardial infarction, unstable angina, liver cirrhosis, infectious colitis, acute hepatitis, ESRD, acute hepatitis A, and diabetic ketoacidosis to have the highest 10 F1-scores in Supplementary Table S6. Thus, a slight difference was observed between the two tree-based ML models in ranking and disease classification. While no significant differences in the predictive power for disease classification were observed between the two boosting models of the tree series, a significant difference was found in predictive power between the two ML models and DL.

### Ensemble model results (DNN, ML)

We developed a new ensemble model by combining our DL model with our two ML models to improve AI performance. We used the validation loss for model optimization. Finally, we created the optimized ensemble model. It achieved an F1-score of 81%, 92% prediction accuracy, 78% precision, and 88% recall in Supplementary Table S7. Compared to our DL and two ML models, the new ensemble model achieved improved performance in all aspects, including F1-score, accuracy, precision, and recall. It achieved the best F1-score, 81%. To improve accuracy further, we constructed an additional ensemble model based on accuracy, which raised the accuracy to 93% in Supplementary Table S8. The ensemble model showed differences in disease prediction compared to the ML and DL. Using the F1-score criterion, the top 10 diseases were acute hepatitis B, malaria, aplastic anemia, meningitis, acute myocardial infarction, acute pyelonephritis, infectious colitis, alcoholic hepatitis, acute pancreatitis, and ESRD in Table 2. Similarly, the ensemble model optimized for accuracy differed from other models in the prediction of disease categories in Table 3.

**Table 2:** Ensemble model TOP 10 result using F1 score performance measure.

ICD 10 CODE(M)	Disease classification	N of data	Precision	Recall	F1-score	Support
B1B	Acute hepatitis B	11	1	1	1	2
B5	Malaria	15	1	1	1	3
D6	Aplastic anaemia, unspecified	11	1	1	1	2
G0	Meningitis	12	1	1	1	2
I2B	Acute myocardial infarction, unspecified	794	0.99	0.98	0.99	161
N1A	Acute pyelonephritis	578	1	0.94	0.97	124
A0B	Infectious colitis	214	0.95	0.98	0.96	42
K7A	Alcoholic hepatitis	70	0.93	1	0.96	13
K8C	Acute pancreatitis	222	0.96	0.96	0.96	45
N1C	ESRD	193	0.97	0.95	0.96	40

*M* modified.

**Table 3:** Ensemble model TOP 10 result using accuracy performance measure.

ICD 10 CODE(M)	Disease classification	N of data	Precision	Recall	F1-score	Support
B5	Malaria	15	1	1	1	3
K7B	Toxic liver disease with hepatitis	23	1	1	1	5
I2B	Acute myocardial infarction, unspecified	794	1	0.98	0.99	162
I2A	Unstable angina	269	0.98	0.96	0.97	55
K7C	Liver cirrhosis(LC)	195	0.97	0.97	0.97	39
N1C	ESRD	193	0.97	0.97	0.97	39
A0B	Infectious colitis	214	0.95	0.98	0.96	42
K8C	Acute pancreatitis	222	0.96	0.96	0.96	45
N1A	Acute pyelonephritis	578	1	0.92	0.96	126
A1A	Pulmonary Tbc	116	0.91	1	0.95	21

*M* modified.

### Confusion matrix of the ensemble model (optimal accuracy model)

**Figure 1:**
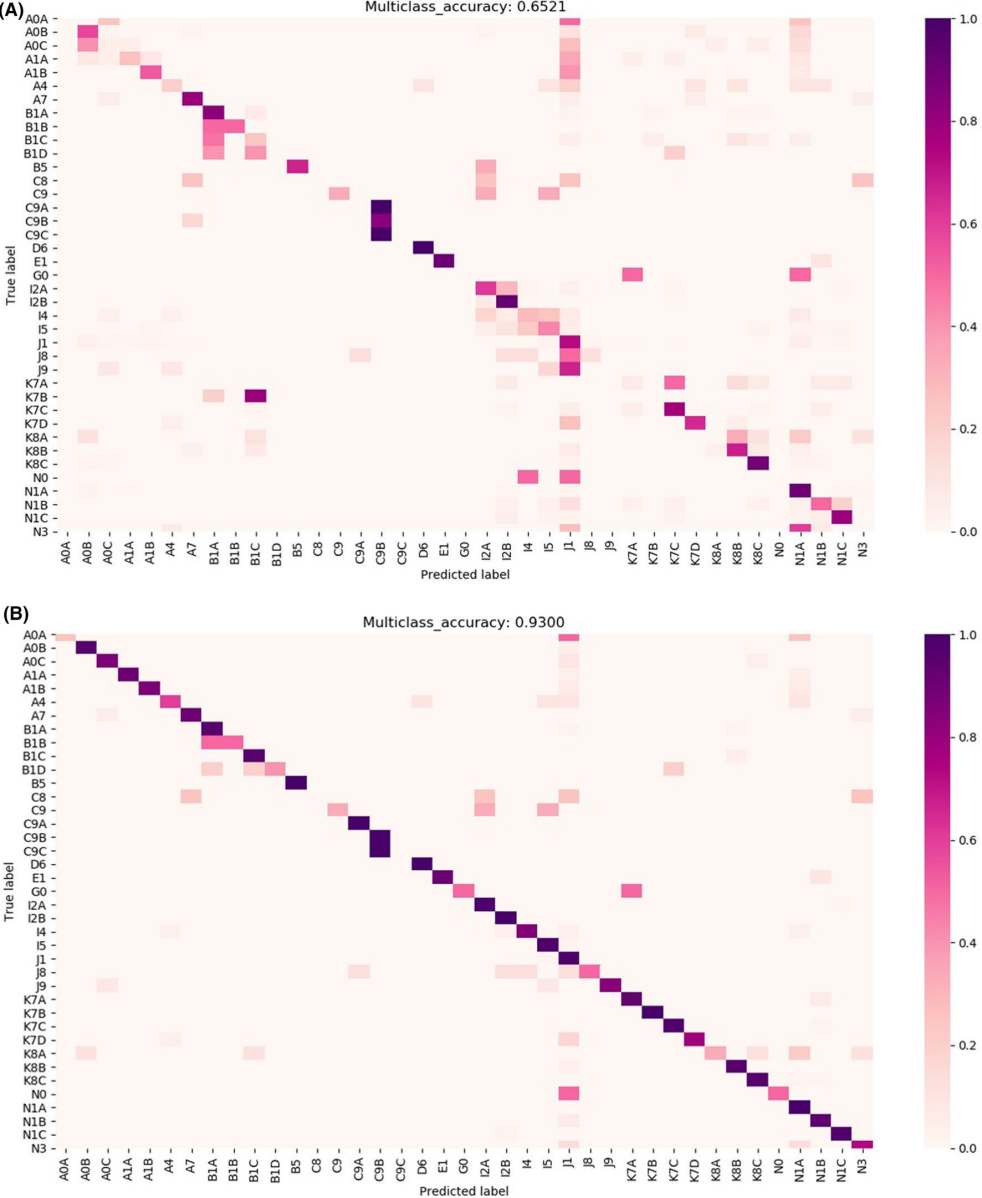
Confusion matrix of the ensemble model (optimal accuracy model): (**A**) The predictive power (accuracy) of TOP1 (representing the most likely disease) result, (**B**) TOP5 (the five most likely diseases) result.

We used confusion matrices for the ensemble model optimized by accuracy. The horizontal axis in Fig. 1 is predicted diseases and the vertical axis is true diseases. Specific label disease classifications were described in Supplementary Table S9. The predictive power (accuracy) of TOP1 (representing the most likely disease) and TOP5 (the five most likely diseases) in the ensemble model was 65% and 93%, respectively in Fig. 1A,B. Specifically, the TOP1 accuracy of the ensemble model was greater than 60% for a large number of diseases. The diseases with accuracies greater than 70% were A7 (scrub typhus), B1A (acute hepatitis A), C9B (acute myeloid leukemia), D6 (aplastic anemia, unspecified), E1 (DKA), I2B (acute myocardial infarction, unspecified), J1 (pneumonia, unspecified), LC (liver cirrhosis), K7D (liver abscess), K8B (cholangitis), K8C (acute pancreatitis), N1A (acute pyelonephritis), and N1C (ESRD). In particular, acute hepatitis and chronic hepatitis tended to be assigned to B1A (acute hepatitis A) by the TOP1 accuracy ensemble model. In addition, several other diseases tended to be predicted as J1 (pneumonia, unspecified). Using the TOP5 accuracy ensemble model, overall disease prediction was very high, with accuracy of 93%. For most diseases, we observed high accuracy, with the model achieving improved prediction accuracy for identifying acute hepatitis and chronic hepatitis as B1A (acute hepatitis A), B1B (acute hepatitis B), B1C (other specified acute viral hepatitis), or B1D (chronic hepatitis B, active). In addition, the TOP5 accuracy ensemble model exhibited improved accuracy for classifying pneumonia, unspecified (J1) compared to the TOP1 accuracy ensemble model. However, non-Hodgkin lymphoma, unspecified (C8) and acute leukemia of unspecified cell type (C9C) were among 39 disease categories that were not predicted at all.

### Differences in the predictive power for disease classification by DL and ML

Compared to two ML models (LightGBM, XGBoost), the DNN model showed higher precision, recall, and F1-score values for sepsis (A4), scrub typhus (A7), acute hepatitis A (B1A), other specified acute viral hepatitis (B1C), acute respiratory distress disease (J8), liver abscess (K7D), and UTI (N3). Meanwhile, the ML models showed higher scores for infectious colitis (A0B), pulmonary tuberculosis (A1A), unstable angina (I2A), congestive heart failure (I5), pneumonia, unspecified (J1), acute respiratory failure (J9), liver cirrhosis (LC), cholangitis (K8B), acute pancreatitis (K8C), acute renal failure (N1B), and ESRD (N1C).

### Feature importance in ML (LightGBM, XGBoost)

The two ML models are similar tree series boosting models, but had differences in feature importance. In LightGBM, the important features were CP (C-reactive protein, CRP), LD (lactate dehydrogenase), GP (γ-glutamyl transpeptidase, γ-GTP), AL (alanine transaminase, ALT), PL (platelet). In the XGBoost model, the important features were CM (CK-MB, creatine kinase-myocardial band isoenzyme), WM (white blood cell in urine by microscopic examination), ALN (ALT/ALT_normal), S (sex), PB (pro-brain natriuretic peptide, Pro BNP), in order of importance. In addition, the Pusan National University team performed feature extraction differently from XGBoost, using a different number of attributes, and the results are shown in Supplement table S10. These results show that even within the same XGBoost ML model, the ranking of feature importance differed according to differences in the engineering process.

### Evaluation of the average impact on model performance of using the mean SHAP value (correlations between feature importance and individual diseases)

**Figure 2:**
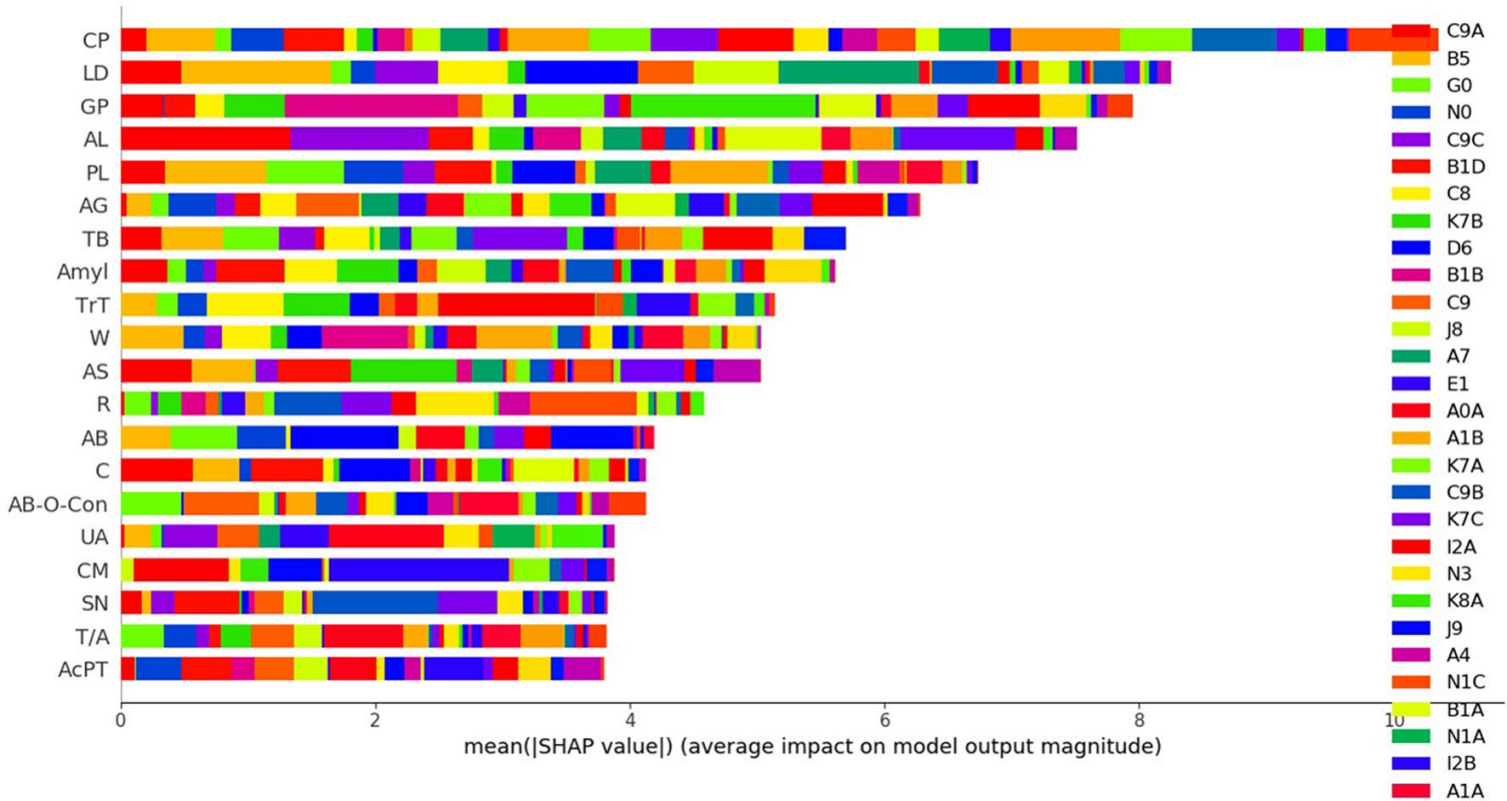
The mean SHAP method result between parameters and disease classifications in LightGBM.

**Figure 3:**
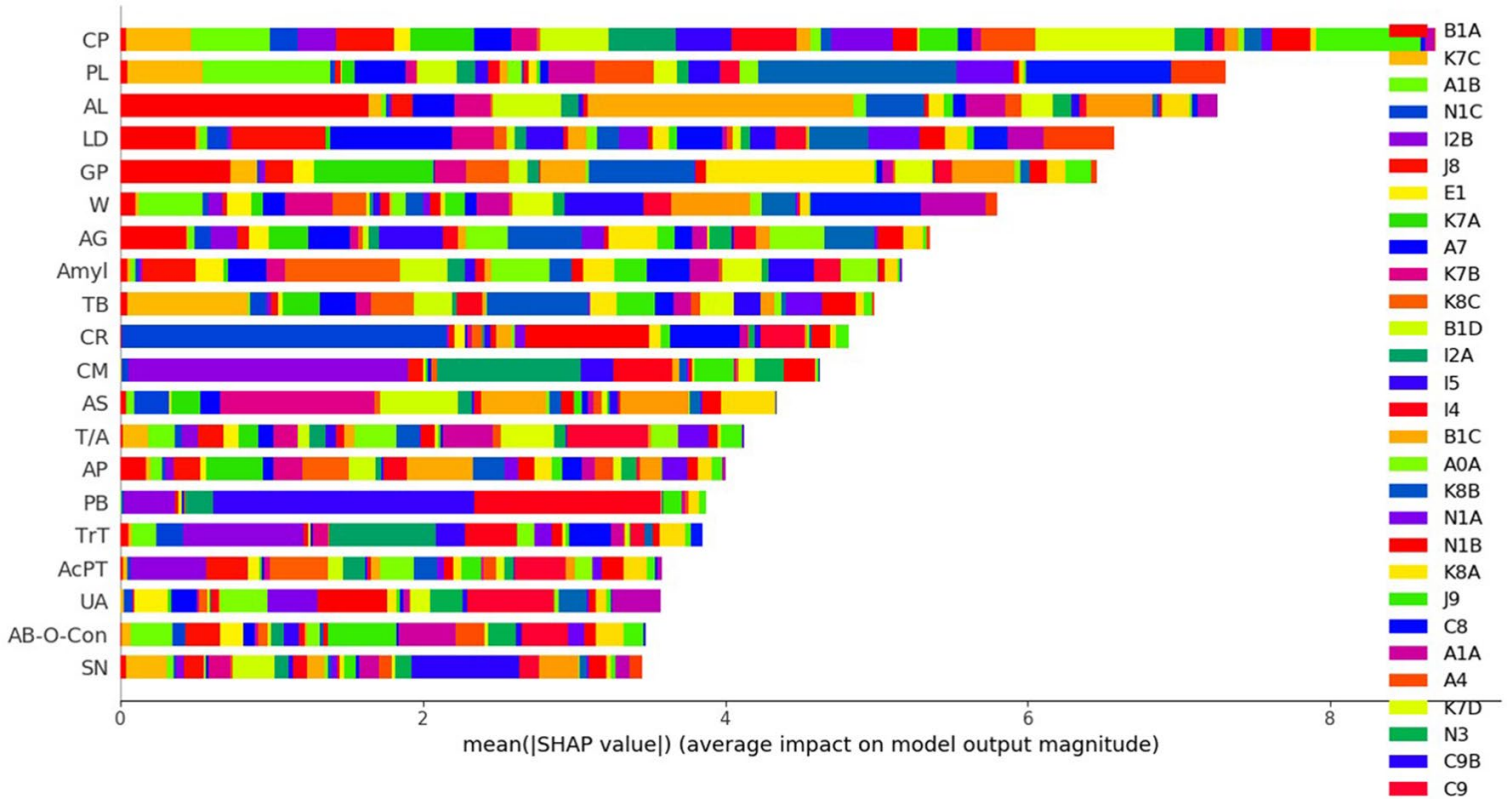
The mean SHAP method result between parameters and disease classifications in XGBoost.

Each disease has a unique ICD-10 code. While the feature importance method (analysis or coding) simply indicates the importance of a given feature (parameter), the mean SHAP method allows the effect of the features on classification (specific disease) to be calculated. We analyzed the mean SHAP values of the two ML models to determine how many features (laboratory tests) correlated with diseases (Figs. 2 and 3). For LightGBM, the TOP10 features with the highest predictive power among the 88 features tested were in the order of CP, LD, GP (γ-GTP), AL, PL, AG (age), TB (total bilirubin), Amyl (amylase), TrT (troponin-T), and W (white blood cell count). In particular, LD and GP were associated with malaria (B5) and acute hepatitis B (B1B), respectively (Supplementary Table S1, S9 and Fig. 2). For XGBoost, CP, PL, AL, LD, GP, W, AG, Amyl, TB, and CR were the ten most important features. Investigating the associations between diseases and features, we confirmed that acute myocardial infarction (I2B) contributed most to the increased CM level. In addition, increased CR was related to ESRD (N1C). Dilated cardiomyopathy (I4) and congestive heart failure (I5) were strongly associated with PB. AST (aspartate aminotransferase) was associated with toxic liver disease (K7B), while an increase in AL was associated with acute hepatitis A (B1A) (Supplementary Table S1, S9 and Fig. 3). For the DL model, the TOP10 features were CP, AG, CR, PB (Pro BNP), PL, TB, AB-O-Con (ABGA-O2 Content), GP, T/A (total protein/albumin), AB (albumin) using mean SHAP value.

### Comparison of AI models (DNN, ML models, and ensemble model) with physicians

Using 88 laboratory parameters (86 laboratory tests, sex and age) for 39 diseases, we compared the results obtained from both our AI models (ML, DL and ensemble models) with physicians’ performance. The same input data were analyzed by five internal medicine specialists, who were assigned a total of 390 analyses with 78 questions each.

Five different physicians showed mean accuracy of 20% for the TOP1 (most likely disease) disease selection (accuracy range: 15–27%, median value: 19%) while the accuracy of AI models was higher (accuracy of ML XGBoost: 65%, LightGBM: 63%, DL: 61%, ensemble: 65%). Five physicians showed diagnostic mean accuracy of 47% for TOP5 disease selection (accuracy range: 33–54%, median value: 50%). However, the accuracies of XGBoost, LightGBM, DL, and the ensemble were 93%, 91%, 91%, and 93%, respectively, for the TOP5 diseases. Overall, the AI model had better diagnostic accuracy than the physicians. We found that the features that contributed to the physicians answering correctly were CP, Pro-BNP, CM, ALT, and AST, in order of importance.

### ROC curve of the optimized ensemble model

For the optimized ensemble model, the modified ICD-10 codes (ICD 10 CODE(M)) for each disease were described in Supplementary Table S9. We classified each disease into class I (group A: A0A, A0B, A0C, A1A, A1B, A4, A7, group B: B1A, B1B, B1C, B1D, B5), class II (C8, C9, C9A, C9B, C9C, D6, E1, G0, I2A, I2B, I4, I5) and Class III (J1, J8, J9, K7A, K7B, K7C, K7D, K8A, K8B, K8C, N0, N1A, N1B, NAC, N3) based on ICD 10 CODE(M). Using python's scikit-learn library, ROC (receiver operating characteristic) curves were drawn for each disease to measure AUC (area under curve). They were shown in Supplementary Figure 1A, B and C. In Class I, the AUC is higher in group B (AUC range: 0.97–1.0) than in group A (AUC range: 0.87–0.98). Among group A, the lowest AUC disease was sepsis (A4) (AUC: 0.87), and the highest disease was tuberculosis pleurisy (A1B). Among group B, the lowest AUC disease was other specified acute viral hepatitis (B1C) (AUC: 0.97), and the highest AUC disease was malaria (B5) (AUC: 1.0). In class II, the overall AUC results ranged from 0.94 to 1.00. In class III, the results of AUC ranged from 0.89 to 0.99. Among them, ARDS (J8), acute cholecystitis (K8A), and UTI (N3) diseases showed relatively low AUC results. The rest of the class III showed AUC results ranging from 0.93 to 0.99.

## Discussion

In this study, our research has a novel point that we could predict 39 diseases accurately that are relatively commonly observed in patients visiting the emergency room through our model. We applied DL and ML models to laboratory data (features). For DL, we used a neural network with two hidden layers, with Relu as the activation function for the input and hidden layers and Softmax as the activation function for the output layer. We tried to improve performance through a hyperparameter optimization process. In general, deepening the neural network layer caused the serious problem of gradient vanishing. In this study, the use of three or more hidden layers caused gradient vanishing and overfitting problems, such as validation loss, to increase. Our neural net model demonstrated very good performance because all data (features), except for sex, were numerical and we optimized performance through tuning of hyperparameters. In this study, the DL (neural network) model also performed well in a categorical classification problem (disease classification) using structured medical data. Among ML models, we selected the LightGBM and XGBoost ML models because they are the state of the art (SOTA) boosting models that show the best performance for a general classification problem. In general, tree-based ML models are known to show good results for classification^12–14^. In contrast, the support vector machine (SVM) and random forest (RF) models showed poor performance (accuracy of SVM: 57%, RF: 61%). For each ML technique, LightGBM and XGBoost showed better performance when the maximum depth was shallow, i.e., when the patient's laboratory data were relatively uncomplicated. We made various efforts to determine the optimal conditions for DL, and the optimal result was obtained using two hidden layers. This result indicates that the data were relatively simple, and we used the dropout method. In the AI of our DL (neural network) model, we used a batch size of 128 and 10 epochs to cover 5145 cases that included 326,686 laboratory test results, meaning that 5145 cases were divided into 128 units and 10 analyses were performed. To calculate the optimal validation loss, we adopted an early stopping method, which caused calculation to stop if the validation loss value did not improve. In this study, analysis was conducted using a patience value of 10 as an option. We analyzed the test set with the optimal weight and bias values obtained through the validation process for a training dataset. To avoid data loss during validation, we used five-fold validation. After the optimization process above, the optimal weight (w) and bias were calculated and used with the test set. An important characteristic of this dataset was that most of its data were numerical. For numerical data, ML generally shows good performance in computational prediction^4,15^. In this study, we aimed to compare DL (neural net) with ML methods. We found that the easiest way to increase performance of ML is proper model selection considering the dataset. Although the completeness of our dataset varied among the 88 total features (parameters), our dataset had approximately 26% missing values. Some features had more than 50% missing values.

For classification problems, the random forest method, a traditional bagging technique, shows good results^16–18^. However, in this study, the results from that method were worse than those of the two ML (LightGBM and XGBoost), which might have been due to the high frequency of missing values. A random forest can be analyzed only if there are no missing values. Thus, missing values must be replaced with the mean, median, or some other value prior to analysis. However, LightGBM and XGBoost automatically fill in the optimal values to replace missing values to calculate the results in an efficient manner^19^. We replaced missing values in LightGBM and XGBoost with the mean and median values and then analyzed performance, which was poor. The decision of whether to replace the missing values for analysis of a dataset should be made considering the characteristics of the data. In our study, we found that replacing missing values with mean or median values biased the results in ML. With regard to DL, because data could only be analyzed if it lacked missing values, we replaced the missing data with median values in DL.

In the present study, physicians showed very low diagnostic accuracy compared to our AI models. Practically, physicians’ diagnosis of a specific disease is made based on the symptom of the patient, physical examination, variable hospital data including laboratory test, image data (computed tomography, x-ray, MRI) and so on. Addition of a lot of unstructured data such as patients’ symptoms and signs, image data (x-ray or computed tomography findings), and biosignals (heart rate, body temperature, etc.) could result in a better and accurate prediction model. However, in real-world practice, it is difficult to use unstructured data because most of these data are in the form of free text, which is not standardization between the hospitals and there is a lot of missing data. Also, it takes a lot of manpower and computing power to process and handle these unstructured data. However, because the performance of the AI model was better than that of physicians using the same limited laboratory dataset, we have the opportunity to re-examine underutilized laboratory data (features, parameters). In particular, both TOP1 and TOP5 showed huge differences in accuracy between the physicians and ML models, showing that the ML model developed in this study could be very helpful for diagnosing diseases. This study further explored correlations between each feature and specific diseases using the newly developed SHAP value library. Interestingly, we found some generally unknown correlations between features (laboratory tests) and classifications (disease), such as increased LDH in malaria and high ALT in viral hepatitis, which are consistent with the results of previous studies^20–22^. In this study, we not only developed meaningful AI models and compared their performance but also revealed associations between diseases and features from the input data.

Regarding the overfitting problem, we separated about 1029 cases (independent data) from the total cases using python library “train test split”. Because our data set is imbalance data, we randomly and evenly selected each specific disease from the data set using stratify option. Also, for a robust model, k-fold cross validation was used. We compared the classic supervised classification models (SVM and RF) and our models. To overcome the overfitting issue, we used two hidden layers in the DNN model, but if more than three were used, the performance was rather degraded. This is thought to be because hospital laboratory data are numerical data and are not somewhat complicated. The reason why we used the DNN model is that deep learning models learn patterns which the tree model cannot solve, so deep learning actually well predicts different diseases (Table 1, Supplementary Table S5, Supplementary Table S6).

In summary, in the present study, we demonstrated that DL and ML could achieve favorable outcomes for a disease classification problem using hospital data. The application of our AI model to large datasets shed new light on the values of various laboratory tests that were not previously recognized. This study contributes to the development of medical informatics by helping AI to learn new patterns of information beyond determining relationships between laboratory data and diseases.

## Methods

**Figure 4:**
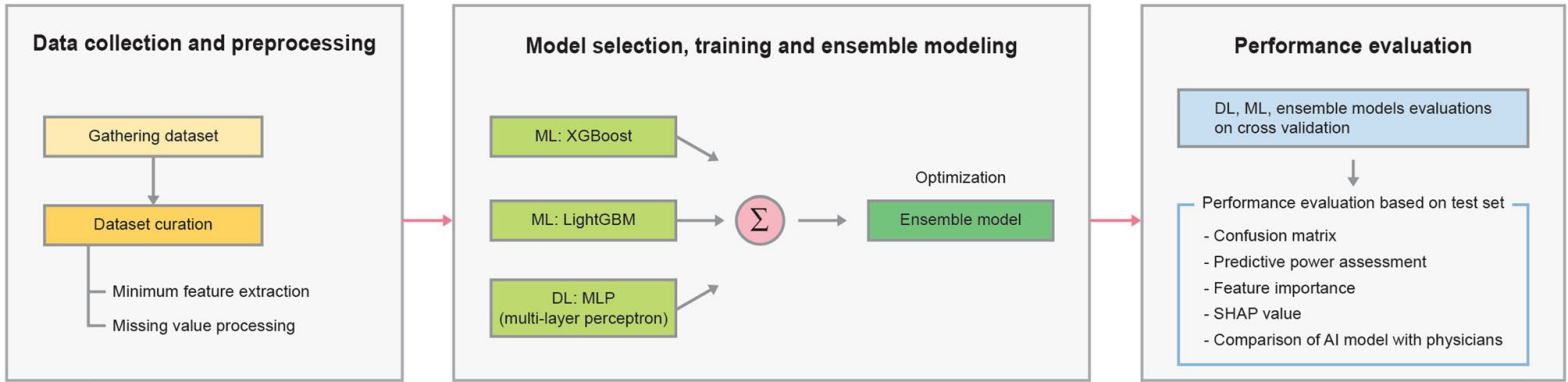
Overall framework of DPMLT. The development of DPMLT methodology involved for three major steps. (1) Data collection and preprocessing (2) Model selection, training and ensemble modeling (3) Performance evaluation.

The principal procedures of the disease prediction model based on laboratory tests (DPMLT) are described in the following sections. The overall workflow of DPMLT is schematically demonstrated in Fig. 4.

### Data collection and preprocessing

We analyzed datasets provided by the Department of Internal Medicine from patients visiting the emergency room and those admitted to Catholic University of Korea St. Vincent’s Hospital in Suwon, Korea, between 2010 and 2019. All patients were at least 19 years old. We collected anonymized laboratory test datasets, including blood and urine test results, along with each patient’s final diagnosis on discharge. This study was approved by the institutional review board of St. Vincent’s Hospital, the Catholic University of Korea, which waived the need of informed consent for this study. All methods and datasets were carried out in accordance with relevant guideline and regulations by the institutional review board. We included data from each patient’s first admission to eliminate any bias caused by previous medications and treatments. We curated the datasets and selected 86 attributes (different laboratory tests) based on value counts, clinical importance-related features, and missing values. We confirmed a total of 88 attributes, including sex and age. Finally, we collected sample datasets of 5145 cases, including 326,686 (73.83%, 326,686/442,470) laboratory test results. For DL, missing values were replaced with the median value for each disease. Finally, 88 selected parameters were used for analysis of the results using DL and ML. In addition, we selected 390 cases covering a total of 39 different classifications (diagnosis), which were evenly divided among five physicians for analysis.

### Feature extraction

Feature extraction plays a major role in the creation of ML models. We performed minimum feature extractions as shown in Supplement 1 (AST/AST normal: aspartate aminotransferase value/50, ALT/ALT normal: alanine aminotransferase value/50, T/A: total protein/albumin).

In this study, we compared the results of DL and ML using structured data and developed a new ensemble model combining DL and ML.

### Model selection and training

#### DL selection

Recently, numerous attempts have been made to utilize DL for bioinformatics. DL has been shown to approximate the function of a complex structure^23^. Another study^5^ reported that DL technologies, including CNN, SAE (stacked auto-encoder), DBN (deep belief network), DNN, and RNN, could be applied to various subjects such as medical images, DNA/RNA, and protein structures. The research in this study was conducted using a DNN for structured data.

#### MLP (multi-layer perceptron)

MLP is a class of DNN^24,25^. An MLP consists of at least three layers: an input layer, a hidden layer, and an output layer. Aside from the input layer, each layer uses a non-linear activation function. This method can analyze data that are not linearly separable. All features used in this study are numeric data except for the ‘sex’ feature. MLP recognizes only numerical data, so we transformed the categorical feature of ‘sex’ into a number using LabelEncoder of the scikit-learn library. MLP does not allow for null values, so we replaced null values with the median value of each feature.

#### Feature normalization and parallelization

Each feature had a different range. However, if the range differs among features, the model may misinterpret the feature range as a real difference, causing it to assign incorrect weights (W) to some features. Therefore, we applied a standard scale to normalize the mean and standard deviation of each feature to (0, 1) by subtracting the mean value of the feature and dividing by its standard deviation value.

#### Hidden layer composition

In our study, the hidden layer was comprised of two layers. We employed the Relu (rectified linear unit) activation function for each layer. We applied the dropout technique to each hidden layer, which is a simple method to prevent overfitting in neural networks^26–28^. Dropout is a method of learning based on random deletion of nodes. It selects and deletes hidden layer nodes during training. The deleted nodes are not forwarded to the next step. During training, whenever data was spilled, nodes were randomly selected for deletion, and all nodes remaining at the time of the test were used. During the test, each node's output was multiplied by the percentage deleted during training.

The activation function of the output layer used the Softmax function to assign a value between 0 and 1 to each class. To train the MLP, we calculated the cross-entropy loss for the difference between the target value and the predicted value. We updated the MLP using the Adam optimizer with a calculated parameter gradient. The Adam optimizer is a method of stochastic optimization^29^ that was introduced as a noise optimizer and is suitable for various objective functions. The optimal model was selected based on low validation set loss. Training was stopped if validation set loss no longer improved after 10 epochs. In our experiments, we implemented this optimization using Keras for the MLP model and scikit-learn for data preprocessing and splitting of datasets.

### ML: boosting model selection

Ensemble modeling is a method of creating strong learners by combining weak learners, and has been widely used for ML recently. Boosting and bagging^30–32^ are the most common ensemble methods. Bagging creates a generalized model through bootstrapping (random sampling) of datasets followed by aggregation into different datasets. Both boosting and bagging are similar learning models based on bootstrapping. However, boosting allows data that were not identified in previous steps to be weighted and classified. Between these methods, we decided to use boosting through the XGBoost and LightGBM algorithms, which are the most popular boosting algorithms. To determine whether performance of boosting was better than that of bagging, and also whether it was vulnerable to overfitting, various hyperparameters of the two algorithms were solved and optimized.

#### XGBoost

The numbers of disease prediction papers using XGBoost with medical data have increased recently^33–36^. XGBoost is an algorithm that overcomes the shortcomings of GBM (gradient boosting machine). The disadvantages of GBM include long learning times and overfitting problems. The most common ways to solve these problems are through parallelization and regularization. Our dataset contained null values, which MLP replaced with the corresponding median values, but XGBoost has a procedure to process null values, so utilized that procedure. The max_depth argument in XGBoost is one factor determining the depth of the decision tree. Setting max_depth to a large number increases complexity and can lead to overfitting. This study found that max_depth was optimally set to 2.

#### LightGBM

Many previous studies have used LightGBM for analysis of medical data^37–40^. LightGBM is a GBM-based model that follows XGBoost. LightGBM is also the most recent algorithm to win an award from Kaggle. The difference between LightGBM and XGBoost is the method by which the tree grows. XGBoost creates a deeper level within the leaf (level-wise/depth-wise), and LightGBM generates a leaf at the same level (leaf-wise). XGBoost uses a level-centered tree-splitting method to keep the tree balanced when it is deepened. LightGBM uses a leaf-centered tree-splitting method to split leaf nodes with the maximum loss value, creating an asymmetric tree. To avoid overfitting in LightGBM, an experiment was conducted by adjusting num_leaves and min_child_samples. Through that experiment, the optimal value of num_leaves was determined to be 2 and that of min_child_samples was 30.

### K-fold cross-validation

In our study, we divided a total of 5145 datasets at a ratio of 8:2 to create the training set and test set. We set the validation data ratio to 0.2 for the training set, which was evaluated using validation loss for model optimization based on the training data.

The number of cases was 5145, which is a relatively small dataset. If the size of the dataset is small, high variance can cause performance problems for the evaluation of the validation dataset. However, if the number of validation data is increased, the number of training data decreases, leading to a problem of high bias. To resolve these trade-offs, the cross-validation method proposed in Sensitivity Analysis of K-Fold Cross-Validation^41–45^ in Prediction Error Estimation was used. We used k-fold cross validation to prevent data loss of the training set.

### SHAP (Shapley Adaptive Explanations)

There are many ways to calculate feature importance. Among them, SHAP value can be seen through ‘Consistent Individualized Feature Attribution for Tree Ensembles’^11^ that have good consistency and accuracy in calculating feature importance. It is a method for the Tree Ensemble model in the experiment of the paper^11^. In our experiment, MLP did not correspond to a Tree ensemble model, so although we didn’t use the same method, we can calculate SHAP value using DeepLIFT^10^.

SHAP is an acronym for Shapley Adaptive Explanations. Relating to the Shapley value, as the name suggests. This Shapley value is a concept in game theory that indicates how much each contributor contributes to a particular outcome. SHAP values provide a strict theoretical improvement by eliminating significant consistency problems.

DeepLIFT^10,46^ is a method for decomposing the output prediction of a neural network on a specific input by backpropagating the contributions of all neurons in the network to every feature of the input. DeepLIFT compares the activation of each neuron to its ‘reference activation’ and assigns contribution scores according to the difference.

### Performance measures

We used the F1 score, accuracy and ROC curve for performance measures.

## Supplementary Information

Supplementary Figure 1A.

Supplementary Figure 1B.

Supplementary Figure 1C.

Supplementary Table 1.

Supplementary Table 2.

Supplementary Table 3.

Supplementary Table 4.

Supplementary Table 5.

Supplementary Table 6.

Supplementary Table 7.

Supplementary Table 8.

Supplementary Table 9.

Supplementary Table 10.
