# Supplementary material for: Development of machine learning model for diagnostic disease prediction based on laboratory tests
Source: Sci Rep. 2021 Apr 7;11:7567. doi: 10.1038/s41598-021-87171-5 (PMC8026627; doi:10.1038/s41598-021-87171-5)
Supplement: Supplementary file 1 — Supplementary Figure 1A. [file 41598_2021_87171_MOESM1_ESM.docx]

Supplementary Fig.1. ROC (receiver operating characteristic) curve of the optimized ensemble model to 39 each specific disease and AUC (area under curve) results

(A)


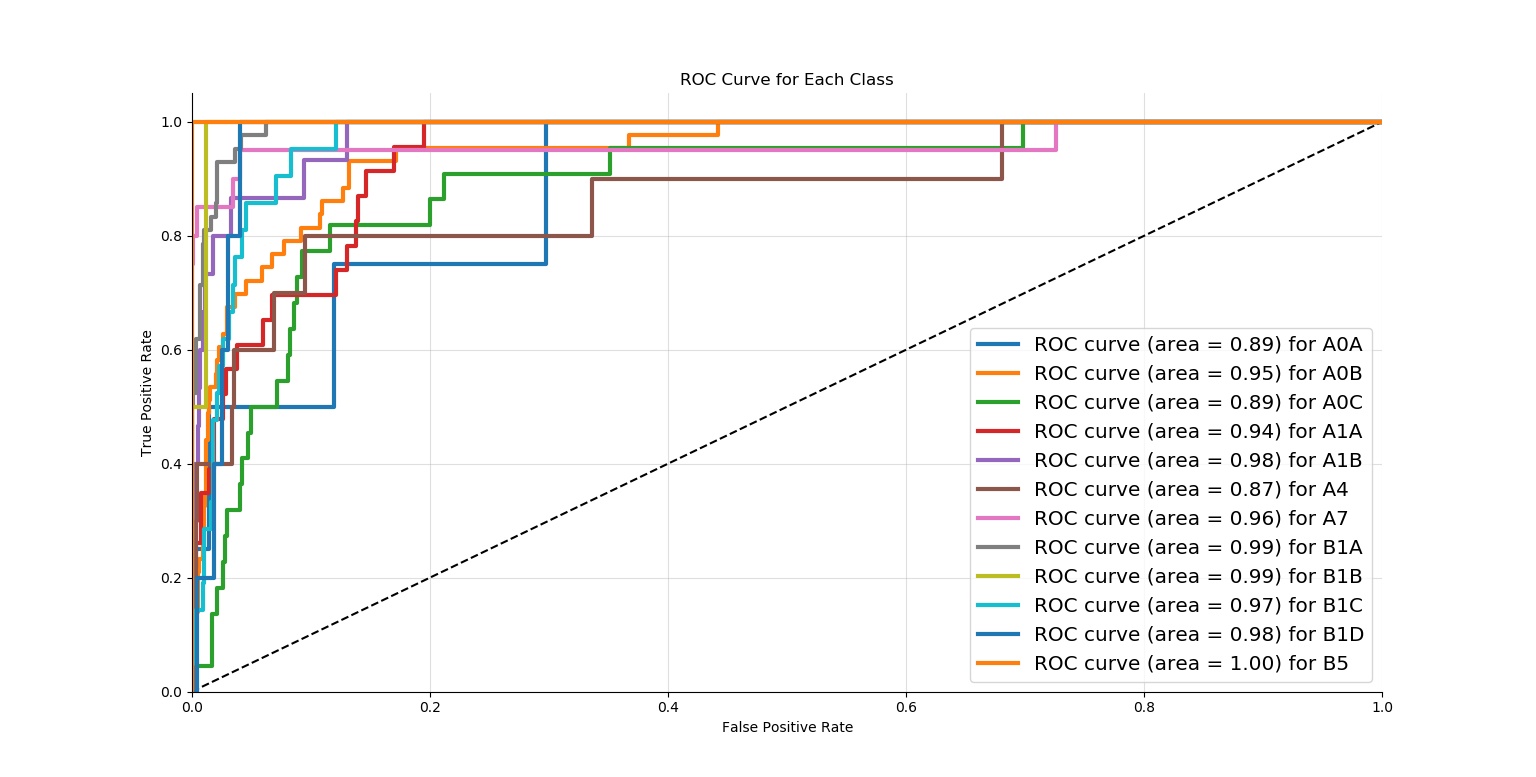


article title**:** Development of Machine Learning Model for Diagnostic Disease Prediction Based on Laboratory Tests

author list: Dong Jin Park, Min Woo Park, Homin Lee, Young-Jin Kim, Yeongsic Kim and Young Hoon Park
