# Supplementary material for: Development of machine learning model for diagnostic disease prediction based on laboratory tests
Source: Sci Rep. 2021 Apr 7;11:7567. doi: 10.1038/s41598-021-87171-5 (PMC8026627; doi:10.1038/s41598-021-87171-5)
Supplement: Supplementary file 4 — Supplementary Table 1. [file 41598_2021_87171_MOESM4_ESM.docx]

Supplementary Table S1. 88 parameters and abbreviation in this study.

| Parameter | Abbreviation |
| --- | --- |
| Sex | S |
| Age | AG |
| WBC Count | W |
| RBC Count | R |
| Hemoglobin | H |
| Hematocrit | Ht |
| Platelet count | PL |
| Seg.-neutrophils | SN |
| Band-neutrophils | BN |
| Lymphocytes | LY |
| Monocytes | MO |
| Eosinophils | EO |
| Basophils | BA |
| ANC (absolute neutrophil count) | AN |
| Nucleated RBC | NR |
| Metamyelocytes | Mt |
| Myelocytes | My |
| Promyelocytes | Pr |
| Atypical lymphocyte | AtL |
| Blasts formed cells | Bl |
| Blast formed cells ratio | Br |
| MCV | MV |
| MCH | MH |
| MCHC | MC |
| Activated PTT Test | AcPT |
| PT Test | PT |
| PT ( )% of normal | PTP |
| INR | IR |
| Glucose | G |
| Total Bilirubin | TB |
| AST (aspartate aminotransferase) | AS |
| AST/AST_normal (50) | ASN |
| ALT (alanine aminotransferase) | AL |
| ALT/ALT_normal (50) | ALN |
| Amylase | Amyl |
| Urea Nitrogen | BU |
| Creatinine | CR |
| Na | N |
| K | K |
| Cl | C |
| CRP (C-Reactive Protein), quan. | CP |
| Glucose(Urine) | G-U |
| Total Protein | PRT |
| Albumin | AB |
| Total protein/Albumin | T/A |
| LDH | LD |
| CPK | PK |
| Leukocyte (urine) | LeU |
| PH | PH |
| Specific Gravity | SP |
| Protein (urine) | PU |
| Ketone | KeU |
| Occult Blood (urine) | OBU |
| Nitrite | NT |
| Urobilinogen | UB |
| Bilirubin (urine) | BilU |
| WBC (urine microscopy) | WM |
| RBC (urine microscopy) | RM |
| ABGA-PH (POCT) | AB-PH |
| ABGA-PCo2 (POCT) | AB-CO |
| ABGA-PO2 (POCT) | AB-O |
| ABGA-HCO3 (POCT) | AB-HC |
| ABGA-Base Excess (POCT) | AB-BA |
| ABGA-O2 Content (POCT) | AB-O-Con |
| ABGA-O2 SAT (POCT) | AB-O-Sat |
| ABGA-TCO2 (POCT) | AB-TCO |
| Troponin-T | TrT |
| Pro BNP (Brain Natriuretic Peptide) | PB |
| CK-MB | CM |
| Ammonia | Amm |
| Alkaline Phosphatase | AP |
| γ-GTP | GP |
| HBs Ag (High Quality) | BAG |
| HBs Ab (High Quality) | BAB |
| Anti-HCV Ab (High Quality) | CAB |
| Direct Bilirubin | DB |
| Uric acid | UA |
| Lipase | LP |
| Phosphorus | P |
| Calcium | CA |
| Magnesium | M |
| Lactic acid (POCT) | LA-P |
| Ferritin | FE |
| Osmo | OS |
| Total Cholesterol | TC |
| HDL-Cholesterol | HDC |
| LDL-Cholesterol | LC |
| Triglyceride | TR |

article title**:** Development of Machine Learning Model for Diagnostic Disease Prediction Based on Laboratory Tests

author list: Dong Jin Park, Min Woo Park, Homin Lee, Young-Jin Kim, Yeongsic Kim and Young Hoon Park
