# Supplementary material for: Development of machine learning model for diagnostic disease prediction based on laboratory tests
Source: Sci Rep. 2021 Apr 7;11:7567. doi: 10.1038/s41598-021-87171-5 (PMC8026627; doi:10.1038/s41598-021-87171-5)
Supplement: Supplementary file 5 — Supplementary Table 2. [file 41598_2021_87171_MOESM5_ESM.docx]

|  | precision | recall | f1-score | Accuracy (TOP1) | Accuracy (TOP5) |
| --- | --- | --- | --- | --- | --- |
| macro avg | 0.77 | 0.87 | 0.8 | 0.609329 | 0.905734 |
| weighted avg | 0.93 | 0.9 | 0.91 | - | - |

Supplementary Table S2. Deep learning model performance result

article title**:** Development of Machine Learning Model for Diagnostic Disease Prediction Based on Laboratory Tests

author list: Dong Jin Park, Min Woo Park, Homin Lee, Young-Jin Kim, Yeongsic Kim and Young Hoon Park
