# Supplementary material for: Development of machine learning model for diagnostic disease prediction based on laboratory tests
Source: Sci Rep. 2021 Apr 7;11:7567. doi: 10.1038/s41598-021-87171-5 (PMC8026627; doi:10.1038/s41598-021-87171-5)
Supplement: Supplementary file 9 — Supplementary Table 6. [file 41598_2021_87171_MOESM9_ESM.docx]

Supplementary Table S6. XGB model TOP 10 result using F1 score performance measure.

| ICD 10 CODE(M) | Disease Classification | N of data | precision | Recall | f1-score | support |
| --- | --- | --- | --- | --- | --- | --- |
| B5 | Malaria | 15 | 1 | 1 | 1 | 3 |
| K7B | Toxic liver disease with hepatitis | 23 | 1 | 1 | 1 | 5 |
| I2B | Acute myocardial infarction, unspecified | 794 | 1 | 0.98 | 0.99 | 162 |
| I2A | Unstable angina | 269 | 0.98 | 0.96 | 0.97 | 55 |
| K7C | Liver cirrhosis(LC) | 195 | 0.97 | 0.97 | 0.97 | 39 |
| A0B | Infectious colitis | 214 | 0.95 | 0.98 | 0.96 | 42 |
| K8C | Acute pancreatitis | 222 | 0.96 | 0.96 | 0.96 | 45 |
| N1C | ESRD | 193 | 0.95 | 0.97 | 0.96 | 38 |
| B1A | Acute hepatitis A | 208 | 0.95 | 0.95 | 0.95 | 42 |
| E1 | DKA | 57 | 0.91 | 1 | 0.95 | 10 |

article title**:** Development of Machine Learning Model for Diagnostic Disease Prediction Based on Laboratory Tests

author list: Dong Jin Park, Min Woo Park, Homin Lee, Young-Jin Kim, Yeongsic Kim and Young Hoon Park
