# Supplementary material for: Development of machine learning model for diagnostic disease prediction based on laboratory tests
Source: Sci Rep. 2021 Apr 7;11:7567. doi: 10.1038/s41598-021-87171-5 (PMC8026627; doi:10.1038/s41598-021-87171-5)
Supplement: Supplementary file 10 — Supplementary Table 7. [file 41598_2021_87171_MOESM10_ESM.docx]

|  | precision | recall | f1-score | Accuracy (TOP1) | Accuracy (TOP5) |
| --- | --- | --- | --- | --- | --- |
| macro avg | 0.78 | 0.88 | 0.81 | 0.646259 | 0.924198 |
| weighted avg | 0.94 | 0.92 | 0.93 | - | - |

Supplementary Table S7. Ensemble model performance result using F1 score

article title**:** Development of Machine Learning Model for Diagnostic Disease Prediction Based on Laboratory Tests

author list: Dong Jin Park, Min Woo Park, Homin Lee, Young-Jin Kim, Yeongsic Kim and Young Hoon Park
