# Supplementary material for: Development of machine learning model for diagnostic disease prediction based on laboratory tests
Source: Sci Rep. 2021 Apr 7;11:7567. doi: 10.1038/s41598-021-87171-5 (PMC8026627; doi:10.1038/s41598-021-87171-5)
Supplement: Supplementary file 12 — Supplementary Table 9. [file 41598_2021_87171_MOESM12_ESM.docx]

Supplementary Table S9. Modified ICD 10 CODE and disease classification

| ICD 10 CODE | Disease | ICD 10 CODE (M) |
| --- | --- | --- |
| A047 | Pseudomembranous colitis | A0A |
| A090 | Infectious colitis | A0B |
| A099 | Acute gastroenteritis | A0C |
| A162 | Pulmonary Tbc | A1A |
| A165 | Tbc pleurisy | A1B |
| A419 | Sepsis | A4 |
| A753 | Scrub typhus | A7 |
| B159 | Acute hepatitis A | B1A |
| B169 | Acute hepatitis B | B1B |
| B178 | Other specified acute viral hepatitis | B1C |
| B181 | Chronic hepatitis B, active | B1D |
| B54 | Malaria | B5 |
| C829 | Non-Hodgkin lymphoma, unspecified | C8 |
| C900 | Multiple myeloma | C9 |
| C910 | Acute lymphoblastic leukemia | C9A |
| C920 | Acute myeloid leukemia | C9B |
| C950 | Acute leukemia of unspecified cell type | C9C |
| D619 | Aplastic anaemia, unspecified | D6 |
| E141 | DKA | E1 |
| G039 | Meningitis | G0 |
| I200 | Unstable angina | I2A |
| I219 | Acute myocardial infarction, unspecified | I2B |
| I420 | Dilated cardiomyopathy | I4 |
| I500 | Congestive heart failure | I5 |
| J189 | Pneumonia, unspecified | J1 |
| J80. | ARDS | J8 |
| J960 | Acute respiratory failure | J9 |
| K701 | Alcoholic hepatitis | K7A |
| K716 | Toxic liver disease with hepatitis | K7B |
| K746 | Liver cirrhosis(LC) | K7C |
| K750 | Liver abscess | K7D |
| K810 | Acute cholecystitis | K8A |
| K830 | Cholangitis | K8B |
| K859 | Acute pancreatitis | K8C |
| N049 | Nephrotic syndrome | N0 |
| N10. | Acute pyelonephritis | N1A |
| N179 | Acute renal failure | N1B |
| N185 | ESRD | N1C |
| N390 | UTI | N3 |

Abbreviation: M, modified; DKA, diabetic ketoacidosis; ARDS, acute respiratory distress syndrome; ESRD, end-stage renal disease; UTI, urinary tract infection

article title**:** Development of Machine Learning Model for Diagnostic Disease Prediction Based on Laboratory Tests

author list: Dong Jin Park, Min Woo Park, Homin Lee, Young-Jin Kim, Yeongsic Kim and Young Hoon Park
