# Supplementary material for: Development of machine learning model for diagnostic disease prediction based on laboratory tests
Source: Sci Rep. 2021 Apr 7;11:7567. doi: 10.1038/s41598-021-87171-5 (PMC8026627; doi:10.1038/s41598-021-87171-5)
Supplement: Supplementary file 13 — Supplementary Table 10. [file 41598_2021_87171_MOESM13_ESM.docx]

Supplementary Table S10. The 10 most important features in Pusan National University team

| Rank | Feature |
| --- | --- |
| 1 | CKMB-2nd |
| 2 | ADA |
| 3 | Creatinine |
| 4 | sex |
| 5 | C3 |
| 6 | Blastsformedcells |
| 7 | Lipase |
| 8 | IDMS-traceable MDRD |
| 9 | TroponinI |
| 10 | ALT |

article title**:** Development of Machine Learning Model for Diagnostic Disease Prediction Based on Laboratory Tests

author list: Dong Jin Park, Min Woo Park, Homin Lee, Young-Jin Kim, Yeongsic Kim and Young Hoon Park
